# Neurological features of 14q24-q32 interstitial deletion: report of a new case

**DOI:** 10.1186/s13039-015-0196-6

**Published:** 2015-11-24

**Authors:** Francesco Nicita, Marilena Di Giacomo, Orazio Palumbo, Emanuela Ferri, Daniela Maiorani, Federico Vigevano, Massimo Carella, Alessandro Capuano

**Affiliations:** Department of Pediatrics and Child Neuropsychiatry, Child Neurology Division, Umberto I Hospital, Sapienza University, Rome, Italy; U.O.C Anatomia Patologica, AOR Ospedale “San Carlo”, Potenza, Italy; Medical Genetics Unit, IRCCS Casa Sollievo della Sofferenza, San Giovanni Rotondo, Italy; Division of Pediatrics – Ospedale Belcolle- Viterbo, Rome, Italy; Division of Neurology, Bambino Gesù Children’s Hospital, IRCCS, Piazza Sant’Onofrio 4, 00165 Rome, Italy

**Keywords:** 14q, microdeletion, epilepsy, myoclonus, EEG, seizures

## Abstract

**Background:**

Interstitial deletions of the long arm of chromosome 14 involving the 14q24-q32 region have been reported in less than 20 patients. Previous studies mainly attempted to delineate recognizable facial dysmorphisms; conversely, descriptions on neurological features are limited to the presence of cognitive and motor delay, but no better characterization exists.

**Case presentation:**

In this paper we report on a patient with a *de novo* interstitial deletion of 5.5 Mb at 14q24.3-q31.1. The deletion encompasses 84 genes, including fourteen Mendelian genes. He presented with dysmorphic face, developmental delay, paroxysmal non-epileptic events and, subsequently, epilepsy.

**Conclusions:**

The clinical and molecular evaluation of this patient and the review of the literature expand the phenotype of 14q23-q32 deletion syndrome to include paroxysmal non-epileptic events and infantile-onset focal seizures.

## Background

Interstitial deletions of the long arm of chromosome 14 involving the 14q24-q32 region have been reported in less than 20 patients [1–11]. Previous studies on patients with 14q interstitial deletions mainly attempted to delineate recognizable facial dysmorphisms; conversely, descriptions on neurological features are limited to the presence of cognitive and motor delay, but no better characterization exists [9].

In this paper we report on a patient with a *de novo* interstitial deletion at 14q24.3-q31.1. We aim to underlie his neurological features comparing them with those observed in patients carrying similar deletions.

## Case presentation

**Fig. 1:**
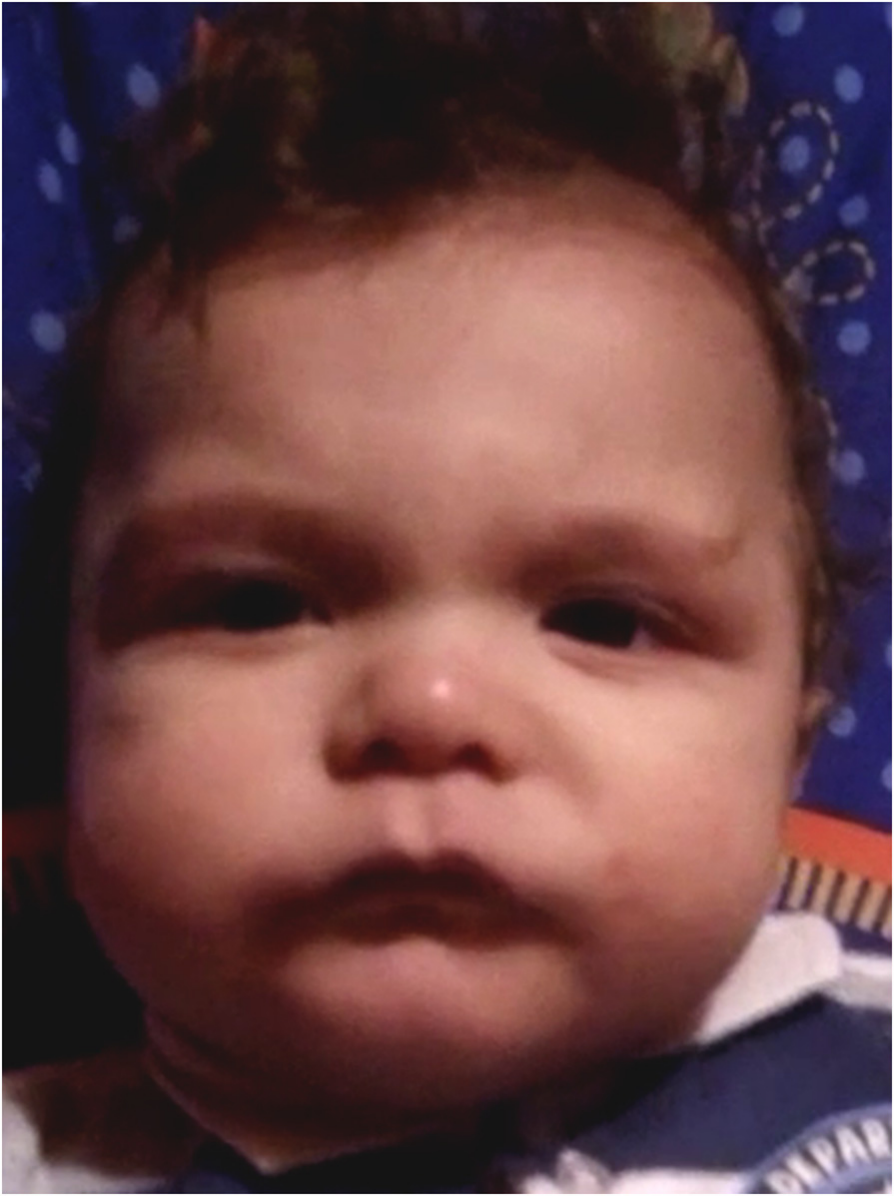
Our patient at 6 months of age: arched eyebrows, down-slanting palpebral fissures, anteverted nostrils, depressed nasal bridge, wide philtrum, and arched thin upper lip

This 2-year-old boy was the third son of non-consanguineous healthy Italian parents. He was born at the fortieth gestational week after an uneventful pregnancy and a spontaneous delivery. Family history was negative for neurological diseases or congenital birth defects. One and five minute Apgar scores were respectively 9 and 10. Birth weight was 3450 g (35–50^th^ percentile), birth length was 55 cm (97^th^ percentile) and head circumference was 33 cm (10^th^ percentile). At birth, bilateral metatarsus varus was evident, requiring conservative orthopedic treatment. No further bone deformities were noticed. He was referred to our attention at age of 8 months for daily, brief episodes of generalized hypertonia and staring. Dysmorphic features of the face were observed, such as arched eyebrows, down-slanting palpebral fissures, anteverted nostrils, depressed nasal bridge, wide philtrum, and arched thin upper lip (Fig. 1). A single cafè-au-lait spot was present on left thigh. Neurological evaluation showed axial hypotonia. Microcephaly was not present. Developmental milestones were mildly delayed: the baby controlled his head at age 5 months, but could not be seated without support. An ictal video-electroencephalogram (EEG) revealed normal findings for age and excluded an epileptic origin of the events. Subsequently, at the age of 9 months, he developed daily episodes of psychomotor arrest, palpebral myoclonias, oral automatisms (e.g., chewing) rarely coupled with vibratory hypertonus. Sleep and awake interictal video-EEGs showed a normal background activity with epileptiform anomalies in bilateral central regions. Therapy with levetiracetam was started and titrated to 40 mg/kg/day. Brain magnetic resonance imaging revealed corpus callosum hypoplasia and enlargement of fronto-temporal sub-arachnoids spaces. Cardiac, abdominal and pelvic (including liver, spleen, gall bladder, pancreas and bladder) ultrasound findings were unremarkable. Eye examination revealed no abnormalities. Routinary biochemical analysis, electrocardiogram, auditory brainstem response and visual evoked potential and electroretinogram yielded normal results. Valproic acid (30 mg/kg/die) and, successively, clonazepam (0.6 mg/die) were added to levetiracetam since daily seizures persisted.

At last follow-up the child is 2 year old. Seizures are controlled by levetiracetam, valproic acid and clonazepam and recurred twice during febrile episodes. Language delay is present: he is able to pronounce 3–5 words. Motor development is improved: he can stand and walk autonomously.

## Materials and methods

Blood was obtained from the proband and his parents after signed informed consent. Genomic DNA was isolated from peripheral blood lymphocytes by using BioRobot EZ1 (Quiagen, Solna, Sweden). DNA concentration and purity were determined with a ND-1000 Spectrophotometer (NanoDrop Technologies, Berlin, Germany), while for the detection of submicroscopic genomic imbalances, we typed genomic DNA by using the Genome-Wide Human SNP 6.0 Array (Affymetrix, Santa Clara, Calif., USA), including 1.8 M oligonucleotide markers, as previously described [12]. Microarrays were washed and stained with the Affymetrix Fluidics Station 450 and scanned with the Affymetrix GeneChip Scanner 3000 using the Command Console software (Affymetrix). Copy number analysis was performed with the Genotyping Console software version 4.1 (Affymetrix) using annotation file version NA32 (hg19) and an in-house reference file consisting of 90 samples. Physical mapping and gene locations were obtained from the University of California Santa Cruz (UCSC, http://genome.ucsc.edu/cgi-bin/hgGateway) Genome Browser, assembly GRCh37.

The SNP array analysis revealed a deletion of approximately 5.5 Mb at 14q24.3-q31.1 region (73,939,744x2,73,939,850-79,446,043x1,79,446,101x2). The deletion was absent in the parents, indicating a *de novo* origin of the rearrangement. The deletion encompasses 84 RefSeq genes (hg19; http://genome.ucsc.edu//).

## Discussion

Deletions of the 14q24-q32 region vary in size (ranging from approximately 10 to 20 Mb) and involve several RefSeq genes resulting in a non-specific dysmorphic appearance associated with neurological involvement of somewhat variable gravity [11]. Attempt to identify a well-detailed genotype-phenotype correlation is hindered by: (1) a few number of published patients; (2) an inaccurate mapping of the deleted segment and lack of detailed breakpoints due to the use of karyotypes or FISH before the era of high-resolution cytogenetic; (3) an association, in previous reviews, of patients with 14q deletions different in size and location; (4) a paucity of data regarding some relevant aspects such as neurological features.

The patient herein reported showed most of the dysmorphic features which have been coupled with the 14q24-q32 deletion, such as hypertelorism, inner epicanthic folds, short and bulbous nose with a depressed bridge, thin upper lip, ears anomalies, pointed chin and micrognathia [11]. No malformations of internal organs were discovered, thus being in line with former reports, which identified heart anomalies in three children [9, 11]. Our case harbored the smallest deletion of the 14q24-q32 region (i.e., 5.5 Mb) reported to date. This region contains several genes whose mutations are known to cause well-defined conditions (Table 1). However, characteristic findings for these entities are currently not present in our patient because most of them are autosomal recessive disorders.

**Table 1:** OMIM genes deleted in our patient, with related phenotypes and model of inheritance

Gene	OMIM	Phenotype	Inheritance
*DNAL1*	610062	Ciliary dyskinesia, primary, 16	AR
*ALDH6A1*	603178	Methylmalonate semialdehyde dehydrogenase deficiency	AR
*VSX2*	142993	Microphthalmia with coloboma;	AR
		Microphthalmia, isolated 2	
*LTBP2*	602091	Glaucoma 3, primary congenital, D;	AR
		Microspherophakia and/or megalocornea, with ectopia lentis and with or without secondary glaucoma;	
		Weill-Marchesani syndrome 3, recessive	
*EIF2B2*	606454	Leukoencephalopathy with vanishing white matter;	AR
		Ovarioleukodystrophy	
*MLH3*	604395	Colorectal cancer, hereditary nonpolyposis, type 7;	AD
		Colorectal cancer, somatic	
*FLVCR2*	610865	Proliferative vasculopathy and hydraencephaly-hydrocephaly syndrome	AR
*TGFB3*	190230	Rienhoff syndrome;	AD
		Arrhythmogenic right ventricular dysplasia 1	
*IFT43*	614068	Cranioectodermal dysplasia 3	AR
*ESRRB*	602167	Deafness, autosomal recessive 35	AR
*POMT2*	607439	Muscular dystrophy-dystroglycanopathy (congenital with brain and eye anomalies), type A, 2;	AR
		Muscular dystrophy-dystroglycanopathy (congenital with mental retardation), type B, 2;	
		Muscular dystrophy-dystroglycanopathy (limb-girdle), type C, 2	
*VIPAS39*	613401	Arthrogryposis, renal dysfunction, and cholestasis 2	AR
*SPTLC2*	605713	Neuropathy, hereditary sensory and autonomic, type IC	AD
*NPC2*	601015	Niemann-Pick disease, type C2	AR

List of *abbreviations*: *AD* autosomal dominant, *AR* autosomal recessive

Clinical picture of our case was primarily dominated by neurological involvement. He presented with hypotonia, mild-to-moderate global developmental and language delay, and paroxysmal episodes of non-epileptic and, successively, epileptic origin. Neurological features of previously described patients are shown in Table 2. Although neurological data have been frequently missed by other authors, mild-to-moderate global developmental delay has been reported in majority of cases and hypotonia has been described in at least 50 % of cases. Additionally, language delay has been reported in 10 out of 14 cases, with four cases able to pronounce no words. Microcephaly was discovered in 50 % of patients. As regards epilepsy, one patient presented a single febrile seizure and another one a single afebrile seizure. Schlade-Bartusiak et al. [8] reported one case with focal epilepsy and temporo-occipital anomalies on EEG. Our patient showed infantile-onset focal epilepsy, which required polytherapy. Notably, epilepsy appeared closely with paroxysmal non-epileptic events. Previously, Ono et al. [6] reported a case of 14q24-q32 deletion and non-epileptic myoclonus status developed at age of 18 months. Although epilepsy and paroxysmal non-epileptic events are both limited to two out of 14 cases (15 %), clinicians, managing patients with 14q24-q32 deletion, should be aware that similar manifestations can appear and require specific investigations, as EEG or neuroimaging, and treatment. However, brain imaging revealed nonspecific findings, such as atrophy, ventricles or subarachnoid spaces enlargement, and corpus callosum hypoplasia, but not neuronal migration disorders.

**Table 2:** Summary of the neurological and cytogenetic features of published patients harboring a 14q23-q32 interstitial deletion

Patient	Sex	Age	Region	Deletion size	Microcephaly	Cognitive delay	Motor delay	Hypotonia	Language delay	Seizures (age of onset; type)	EEG	Brain scan	Sensorineural Deafness	Other neurological findings
1- Turleau et al., 1984 [1] (P1)	M	Died at 2y (cardiac failure?)	14q23-q32	NA	Yes (− 3 SD)	Yes Severe	Yes Severe	NR	Yes	No	Normal	NR	NR	NR
2- Kawamura et al., 1985 [2]	M	11y^a^	14q24.3q32.1	NA	Yes	Yes	Yes	NR	Yes	Single afebrile seizure at 11 y^a^	No epileptic anomalies^a^	NR	NR	NR
3- Yamamoto et al., 1986 [3]	M	10mo – 12y^a^	14q24.3-32.1	NA	Yes (−1.7 SD)	Yes Mild	Yes Mild	NR	No language^a^	NR	Normal	Mild generalized cortical atrophy (CT)	No (ABR)	NR
4- Rivera et al., 1992 [4] (P1)	M	1y 3mo	14q24-q32	NA	Yes (<3 %)	NR	Yes Mild	NR	No words at 15 mo	NR	NR	NR	NR	NR
5- Byth et al. 1995 [5] (P1251)	F	NR	14q23-q32	11.6 MB (76,822,337–88,488,190)^b^	No	Yes Mild	Yes Mild	NR	NR	NR	NR	NR	NR	NR
6- Byth et al. 1995 [5] (P1141)	F	5y	14q24.1-q31	NA	Yes	Yes	No	NR	NR	NR	NR	NR	NR	NR
7- Ono et al., 1999 [6]	M	2y	14q24.3-q32.1	NA	Yes (−2.7 SD)	NR	Yes	No	NR	Simple FS at 13mo;	Spikes in right O region	Frontal atrophy and delayed myelination (MRI)	NR	Non-epileptic myoclonic status at 18mo
8- Le Meuer et al., 2005 [7]	M	Died at 6 mo (cardiac failure)	14q23.3 ≈ 24.2q 31.1	13.9 Mb (67,736,534–81,673,589)^b^	No	Yes	Yes	Yes	NA	NR	Poor modulation	Mild enlargement of the ventricles and the pericerebral spaces (MRI)	Immature response (ABR)	Poor sucking and swallowing, abnormal ocular contact
9- Schlade-Bartusiak et al., 2008 [8] (HSC23984)	F	5y	14q24-3q32.1	18.5 Mb (77,823,431–96,400,270)^b^	No	Yes Mild	Yes Mild-moderate	Yes	No language	2y; Focal	T-O anomalies	Normal (MRI)	Moderate hearing loss within the 1000–4000 Hz frequency range (ABR)	NR
10- Zollino et al., 2009 (P22)	F	19y	14q24.3q32.12	13.9 Mb (77,867,749–91,848,982)^b^	No	Yes	NR	Yes	Yes	No	NR	NR	NR	Motor stereotypies, aggressive during adolescence
11- Zollino et al., 2009 (P25)	M	18y	14q24.3q32.13	20.9 Mb (73,942,355–94,914,188)^b^	Yes	Yes	NR	Yes	Yes	No	NR	NR	NR	Hyperactive, aggressive, hands flapping, echolalia
12- Cingoz et al., 2011	M	10y	14q24.3-q32.2	21.5 Mb (77,226,431–98,771,224)^b^	No	Yes	Yes	Yes	No language	NR	NR	NR	NR	NR
13 – Riegel et al., 2014 [11]	M	6y 9mo	14q24.3-q31.3	13.1 Mb (76,822,337–88,488,190)^b^	No	Yes Mild	Yes Mild	Yes	Yes	NR	Normal	Small area of gliosis in cerebellar region (MRI)	No (ABR)	NR
14- This report	M	2y 1mo	14q24.3-q31.1	5,5 Mb (73,939,850–79,446,043)	No	Yes Mild	Yes Mild	Yes	Yes	9 mo; Focal	Spikes in bilateral C regions	Corpus callosum hypoplasia; enlargement of fronto-temporal sub-arachnoids spaces	No (ABR)	Paroxysmal non epileptic events

List of *abbreviations*: *M* male, *F* female, *y* years, *mo* months, *NA* not applicable, *NR* not reported, *SD* standard deviation, *CT* computed tomography, *MRI* magnetic resonance imaging, *ABR* auditory brainstem response, *T* temporal, *O* occipital, *C* central

^a^Additional information on these two patients were reported by Ono et al. [6] on the basis of personal communications by Kawamura and Yamamoto

^b^Minimal deletion and base pairs are taken from Riegel et al. [11]

**Fig. 2:**
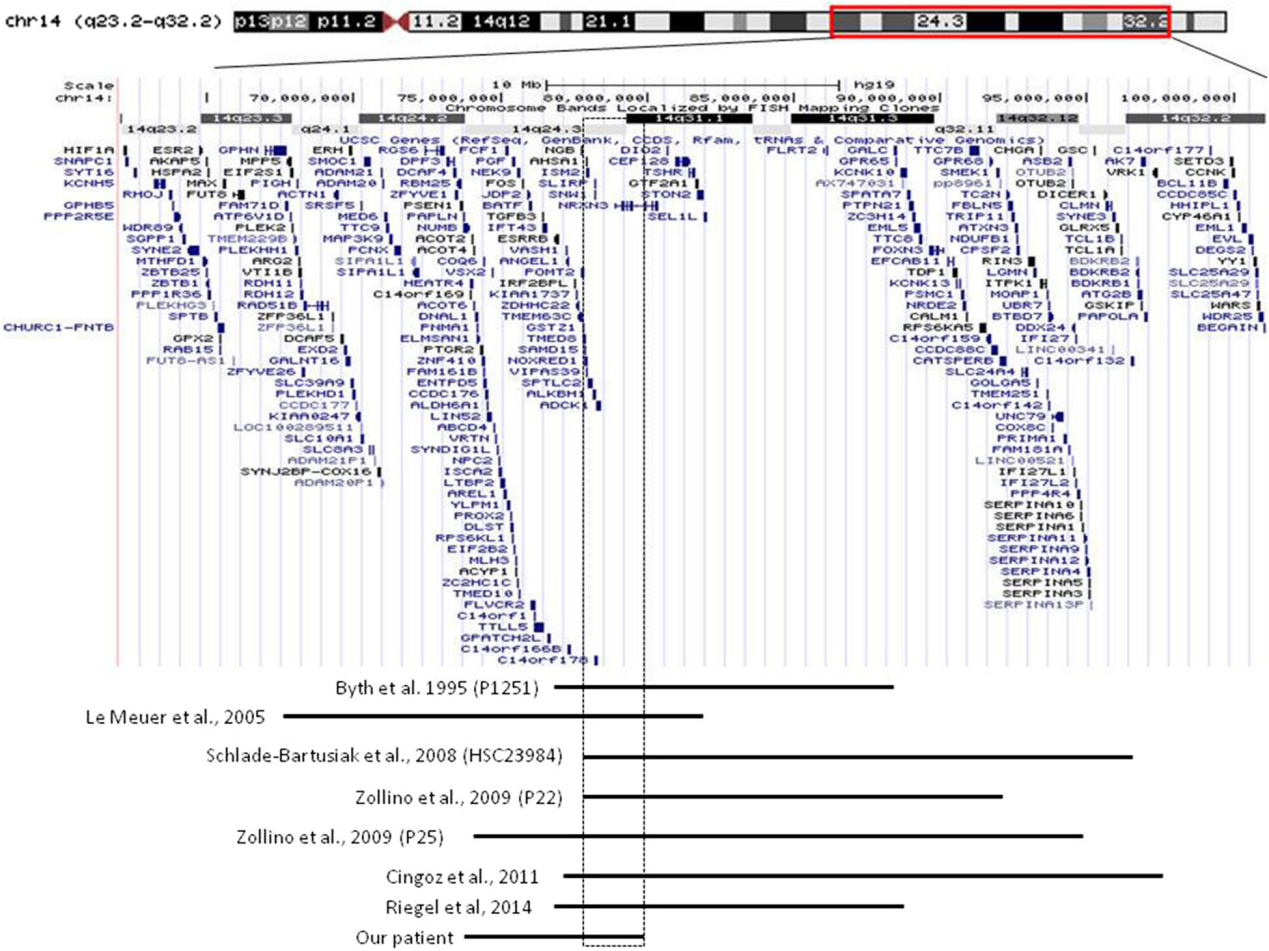
Representation of the 14q23-q32 genomic region (USCS GRCh37/hg19 assembly) and extension of deletion in our case and previously reported patients with known deletion size (minimal deletion and base pairs are taken from Riegel et al. [11] The overlapped deleted region of approximately 1.6 Mb (chr14: 77,823,431–79,446,043) has been marked

From a genetic point of view, the chromosome 14 is involved in several single-gene as well as microdeletion diseases. In fact, patients with ring 14, 14q11-q13 deletion, 14q24.1-q24.3 deletion, 14q24-q32 deletion, and 14q32 deletion have been reported [13]. Currently, a full distinction between these forms is not available due to the few patients reported for each condition, with the exception of ring 14 syndrome. The ring 14 syndrome is characterized by a more severe clinical picture, with postnatal growth delay, microcephaly, minor facial anomalies, eye involvement with retinal degeneration, drug-resistant epilepsy, and cognitive delay with speech problems [10]. Patients with 14q11-q13 deletion have microcephaly, corpus callosum anomalies, gastrointestinal anomalies and, in smaller percentage, vision loss secondary to cortical blindness and/or optic nerve atrophy, seizures, recurrent infections, and genitourinary and renal abnormalities [14]. Deletion of the 14q24.1-q24.3 region has been coupled with congenital heart defects, brachydactyly, mild intellectual disability, and facial dysmorphic signs [15]. Finally, mental retardation, hypotonia, postnatal growth retardation and dysmorphic features have been observed in patients with 14q32 deletion [13]. Previously, other authors attempted to create a deletion map to establish genotype-phenotype correlation in 14q interstitial deletions [9, 10]. In particular, Cingoz and colleagues suggested that haploinsufficiency of Neurexin 3 (*NRXN3*), a protein that function in the nervous system as receptor and cell adhesion molecule and which has been associated with autism, may underlie cognitive and neurological defects [9]. Focusing on the overlapped deleted region in our and other patients with known breakpoints (Fig. 2), we were able to identify a small region of approximately 1.6 Mb (chr14: 77,823,431-79,446,043), which is shared by all eight patients. This region contains the *TMED8*, *SAMD15*, *NOXRED1*, *VIPAS39*, *ISM2*, *AHSA1*, *SPTLC2*, *ALKBH1*, *C14orf178*, *SLIRP*, *SNW1*, *ADCK1*, and part of *NRXN3* genes. Apart from *VIPAS39* and *SPTLC2* – which are associated with a form of arthrogryposis with renal dysfunction and cholestasis, and with hereditary sensory and autonomic neuropathy, type IC (Table 2), respectively – other genes are not known to cause specific phenotypes. Looking for candidate genes, which might influence phenotype of the patients, we focused on *ALKBH1*, a histone H2A dioxygenase that regulates a subset of genes required for neural development in embryonic stem cells [16]. We speculate that haploinsufficiency of *ALKBH1* and *NRXN3* might contribute to neurological involvement in 14q24-q32 deletion.

## Conclusion

In summary, clinical findings of our patient are consistent with those previously reported in cases of 14q24-q32 deletion. These are characterized by dysmorphic features and neurological involvement with hypotonia, global developmental delay, and, in some cases, epilepsy. Analysis of previous cases, with known deletion size, allowed us to identify an overlapped deleted region of 1.6 Mb that contains genes underlying phenotype. A better characterization of these deletions by molecular cytogenetic techniques will help us to make more precise genotype-phenotype correlations. Long term follow-up of patients with these newly described genomic syndromes will give essential information for genetic counseling of patients with similar anomalies.

## Consent

Written informed consent was obtained from the patient’s parents for publication of this Case report and any accompanying images. A copy of the written consent is available for review by the Editor-in-Chief of this journal.
